# Gender Differences in Opioid Use Among Older Adults With Chronic Pain

**DOI:** 10.1093/geroni/igaf122.071

**Published:** 2025-12-31

**Authors:** Sadaf Milani, Yong Shan, Yong-Fang Kuo, Mukaila Raji

**Affiliations:** University of Texas Medical Branch, Galveston, Texas, United States; University of Texas Medical Branch, Galveston, Texas, United States; University of Texas Medical Branch, Galveston, Texas, United States; University of Texas Medical Branch, Galveston, Texas, United States

## Abstract

Women and minoritized individuals often have their pain underdiagnosed and undertreated. Cognitive status adds a layer of complexity, as self-report of pain may be affected as individuals decline. Our objective was to assess self-reported prescription opioid use among older adults, while considering race/ethnicity and cognitive status. We used 2018 data from the Health and Retirement Study linked to Medicare Claims. We included participants with chronic pain and at least one year of continuous enrollment (n = 2,990), excluding those with HMO plans. Our outcome was self-reported past three-month opioid use. Our exposure was gender. Effect modifiers included race/ethnicity (non-Hispanic White, non-Hispanic Black, Hispanic) and cognitive status (no dementia, cognitive impairment no dementia, dementia). We used logistic regression to examine the association of gender with opioid use, controlling for demographic and health characteristics. We tested the interaction of gender with race/ethnicity and cognitive status. Opioid use did not differ between women and men (15.4% vs. 13.6%). There was an interaction between gender and cognitive status as well as race/ethnicity. In gender-stratified models, non-Hispanic Black and Hispanic women had lower odds of opioid use compared to non-Hispanic White women. There were no racial/ethnic differences for men. Lastly, women with dementia had lower odds of opioid use, while men with dementia had higher odds of opioid use compared to their counterparts with normal cognition. This work highlights the complexities of opioid use among older adults. The final version will incorporate data from Medicare Part-D to examine the variation of prescriptions for chronic pain treatment.

